# High triglyceride-glucose index is associated with early recurrent ischemic lesion in acute ischemic stroke

**DOI:** 10.1038/s41598-021-94631-5

**Published:** 2021-07-28

**Authors:** Ki-Woong Nam, Hyung-Min Kwon, Yong-Seok Lee

**Affiliations:** 1 Department of Neurology, Seoul Metropolitan Government-Seoul National University Boramae Medical Center, 20 Boramae-ro 5-gil, Dongjak-gu, Seoul, 07061 South Korea; 2 Department of Neurology, Seoul National University College of Medicine, Seoul, South Korea

**Keywords:** Neuroscience, Neurology, Neurological disorders

## Abstract

The triglyceride-glucose (TyG) index has been associated with various metabolic, cardiovascular, and cerebrovascular diseases. We evaluated the association between the TyG index and early recurrent ischemic lesions (ERILs) in patients with acute ischemic stroke (AIS). We included consecutive patients diagnosed with AIS between 2010 and 2016. ERILs were defined as new diffusion-weighted imaging lesions outside the initial symptomatic lesion area. The TyG index was calculated using the following formula: log scale of fasting triglyceride × fasting glucose/2. A total of 176 patients with AIS were evaluated. In the multivariable analysis, the TyG index remained significant (adjusted odds ratio [aOR] 2.63, 95% confidence interval [CI] 1.34–5.15). This close correlation between the TyG index and ERIL was pronounced in ERIL-same group (aOR 2.84, 95% CI 1.40–5.78), but not in ERIL-different group. When comparing the relationship between the TyG index and ERIL by stroke mechanisms, only the intracranial- and extracranial-large artery atherosclerosis groups showed significantly higher TyG index values in patients with ERIL than those without. In conclusion, a higher TyG index was associated with ERIL, especially ERIL-same, in patients with AIS. The TyG index appears to be involved in ERIL occurrence by a mechanism related to atherosclerosis.

## Introduction

Stroke recurrence increases the risk of subsequent disability and mortality in patients with acute ischemic stroke (AIS)^1^. Therefore, identifying these high-risk groups is an important issue in clinical practice. However, even if well-trained neurologists perform daily neurological examinations, differentiating between initial neurological deficits and clinical worsening in AIS patients is challenging^1–3^; therefore, clinical recurrence after stroke occurrence has been reported to be less than 5%, but this may be underestimated^2^.

New diffusion-weighted imaging (DWI) lesions, also referred to as early recurrent ischemic lesion (ERIL), are frequently observed during the acute period in patients with AIS^1,4^, occurring in up to 40% of patients in the first week^1,5^. Although ERIL is mostly asymptomatic, it is clinically important as it is associated with subsequent stroke, dementia, and vascular death^1,5,6^. In addition, since the symptoms of stroke are determined by the location, size, and number of lesions, ERIL is considered to have a similar pathology as an index stroke or clinical recurrence^5,6^. Therefore, as a surrogate marker of stroke recurrence, studies on risk factors for ERIL have been performed, leading to the discovery of several clinical, laboratory, and radiological risk factors^3,4,6,7^.

The triglyceride-glucose (TyG) index is a marker of chronic insulin resistance (IR) easily obtained using fasting glucose and triglyceride (TG)^8^. Chronic IR is known to affect stroke prognosis, including recurrence, through various metabolic or inflammatory pathways^9–11^. Indeed, the TyG index has recently been found to be closely related to various metabolic, cardiovascular, and cerebrovascular diseases^12–17^. Fasting glucose and TG are well-known risk factors for stroke recurrence^18,19^, and a recent study has reported that the TyG index, also as a comprehensive indicator of acute hyperglycemia and hypertriglyceridemia, is associated with early neurological deterioration (END) in AIS patients^20^.

In this study, we demonstrated the association between the TyG index and ERIL in patients with AIS. Interestingly, previous studies have shown that the incidence of ERIL differs depending on the stroke mechanism^4^. Thus, we also compared the relationship between the TyG index and ERIL according to stroke mechanism.

## Results

A total of 176 patients with AIS were evaluated (mean age, 71 ± 13 years; males, 58.5%; mean initial NIHSS score, 7 ± 7). ERIL was detected in 74 (42.0%) patients: 57 (32.4%) cases of ERIL-same and 17 (9.7%) cases of ERIL-different. The mean time from symptom onset to visit was 1 ± 1 days, and the mean time to follow-up MRI was 3 ± 2 days. The mean TyG index value was 8.48 ± 0.55. Other baseline characteristics are shown in Supplementary Table 1. In this study population, the TyG index was correlated with atrial fibrillation, stroke mechanism, WBC counts, TG/HDL ratio, ERIL, and ERIL-same (Supplementary Table S2).

In the univariate analysis, ERIL was associated with age, initial NIHSS score, fasting glucose, total cholesterol, LDL cholesterol, TG, TG/HDL ratio, TyG index, and recanalization (Table 1). In the multivariable logistic regression analysis, the TyG index (adjusted odds ratio [aOR] 2.63, 95% confidence interval [CI] 1.34–5.15) was positively associated with ERIL after adjusting for confounders (model 1). When TyG index was created as a multi-categorical variable using tertile and analyzed, a positive quantitative relationship was also shown between the two (tertile 2: aOR 4.22, 95% CI 1.71–10.42; tertile 3: aOR 4.37, 95% CI 1.73–11.03, model 2). Age and recanalization were also positively associated with ERIL, independent of the TyG index (Table 2). This close association between the TyG index and ERIL was more pronounced in the ERIL-same group (aOR 2.84, 95% CI 1.40–5.78, Supplementary Table S3). On the contrary, the ERIL-different group was not associated with the TyG index (Supplementary Table S4). Meanwhile, the TG/HDL ratio (aOR 1.21, 95% CI 0.96–1.54) showed only a marginal tendency and was not statistically significant with ERIL (Supplementary Table S5).

**Table 1:** Comparisons of baseline characteristics of patients with and without early recurrent ischemic lesion.

	No ERIL (n = 102)	ERIL (n = 74)	*P* value
Age, years [IQR]	70 [68–73]	74 [70–79]	0.029
Sex, male, n (%)	62 (60.8)	41 (55.4)	0.475
Visit time, days [IQR]	0 [0–1]	0 [0–1]	0.900
Follow-up MRI time, days [IQR]	2 [2–4]	3 [3, 4]	0.233
Hypertension, n (%)	72 (70.6)	50 (67.6)	0.668
Atrial fibrillation, n (%)	32 (31.4)	21 (28.4)	0.669
Smoking, n (%)	34 (33.3)	18 (24.3)	0.196
**Stroke mechanisms, n (%)**			0.115
IC-LAA	19 (18.6)	22 (29.7)	0.085
EC-LAA	23 (22.5)	21 (28.4)	0.378
Cardioembolic	35 (34.3)	21 (28.4)	0.404
Cryptogenic	25 (24.5)	10 (13.5)	0.071
Initial NIHSS score [IQR]	4 [2–11]	6 [3–15]	0.006
Systolic BP, mmHg [IQR]	154 [149–160]	148 [143–158]	0.718
Diastolic BP mm Hg [IQR]	82 [79–84]	85 [83–91]	0.111
HbA1c, % [IQR]	5.9 [5.8–6.1]	5.8 [5.7–6.1]	0.365
Fasting glucose, mg/dL [IQR]	97 [95–103]	102 [99–111]	0.043
Total cholesterol, mg/dL [SD]	175 [169–186]	185 [177–197]	0.066
LDL cholesterol, mg/dL [IQR]	100 [89–110]	113 [102–123]	0.045
HDL cholesterol, mg/dL [SD]	43 [39–46]	41 [40–46]	0.572
Triglyceride, mg/dL [IQR]	80 [73–94]	88 [83–113]	0.015
White blood cell, × 10^3^/uL [IQR]	7.56 [6.78–8.53]	7.94 [6.93–8.63]	0.581
High-sensitivity CRP, mg/dL [IQR]	0.30 [0.17–0.47]	0.19 [0.10–0.41]	0.769
TG/HDL ratio [IQR]	1.95 [1.70–2.23]	2.33 [1.88–2.79]	0.049
TyG index [IQR]	8.32 [8.21–8.50]	8.51 [8.44–8.70]	0.002
Recanalization, n (%)	14 (13.7)	20 (27.0)	0.027

*ERIL* early recurrent ischemic lesion, *MRI* magnetic resonance imaging, *LAA* large artery atherosclerosis, *NIHSS* National Institutes of Health Stroke Scale, *BP* blood pressure, *LDL* low-density lipoprotein, *HDL* high-density lipoprotein, *CRP* C-reactive protein, *TG* triglyceride, *TyG index* triglyceride-glucose index.

**Table 2:** Multivariable logistic regression analysis of possible predictors for early recurrent ischemic lesion.

	Crude OR (95% CI)	*P*-value	Adjusted OR (95% CI)	*P*-value
Model 1*				
Age	1.03 [1.01–1.05]	0.020	1.04 [1.01–1.07]	0.008
Stroke mechanism		0.121		0.102
IC-LAA	2.90 [1.11–7.53]	0.029	2.15 [0.75–6.11]	0.152
EC-LAA	2.28 [0.89–5.86]	0.086	2.20 [0.78–6.20]	0.136
Cardioembolic	1.50 [0.60–3.73]	0.383	0.77 [0.27–2.21]	0.623
Cryptogenic	Ref	Ref	Ref	Ref
Initial NIHSS score	1.06 [1.01–1.11]	0.013	1.05 [1.00–1.11]	0.044
Recanalization	2.33 [1.09–4.99]	0.030	4.23 [1.67–10.74]	0.002
TyG index	2.32 [1.29–4.16]	0.005	2.63 [1.34–5.15]	0.005
**Model 2**^†^				
Age	1.03 [1.01–1.05]	0.020	1.04 [1.01–1.07]	0.008
Stroke mechanism		0.121		0.059
IC-LAA	2.90 [1.11–7.53]	0.029		0.127
EC-LAA	2.28 [0.89–5.86]	0.086		0.054
Cardioembolic	1.50 [0.60–3.73]	0.383		0.745
Cryptogenic	Ref	Ref	Ref	Ref
Initial NIHSS score	1.06 [1.01–1.11]	0.013		0.056
Recanalization	2.33 [1.09–4.99]	0.030		0.001
TyG index		0.003		0.002
Tertile 1 (< 8.24)	Ref	Ref	Ref	Ref
Tertile 2 (8.24–8.63)	3.11 [1.41–6.83]	0.005	4.22 [1.71–10.42]	0.002
Tertile 3 (> 8.63)	3.69 [1.67–8.14]	0.001	4.37 [1.73–11.03]	0.002

*IC-LAA* intracranial large artery atherosclerosis, *EC-LAA* extracranial large artery atherosclerosis, *NIHSS* National Institutes of Health Stroke Scale, *BP* blood pressure, *TyG index* triglyceride-glucose index.

*TyG index was introduced as a continuous variable.

^†^TyG index was introduced as a categorical variable based on tertile.

**Figure 1:**
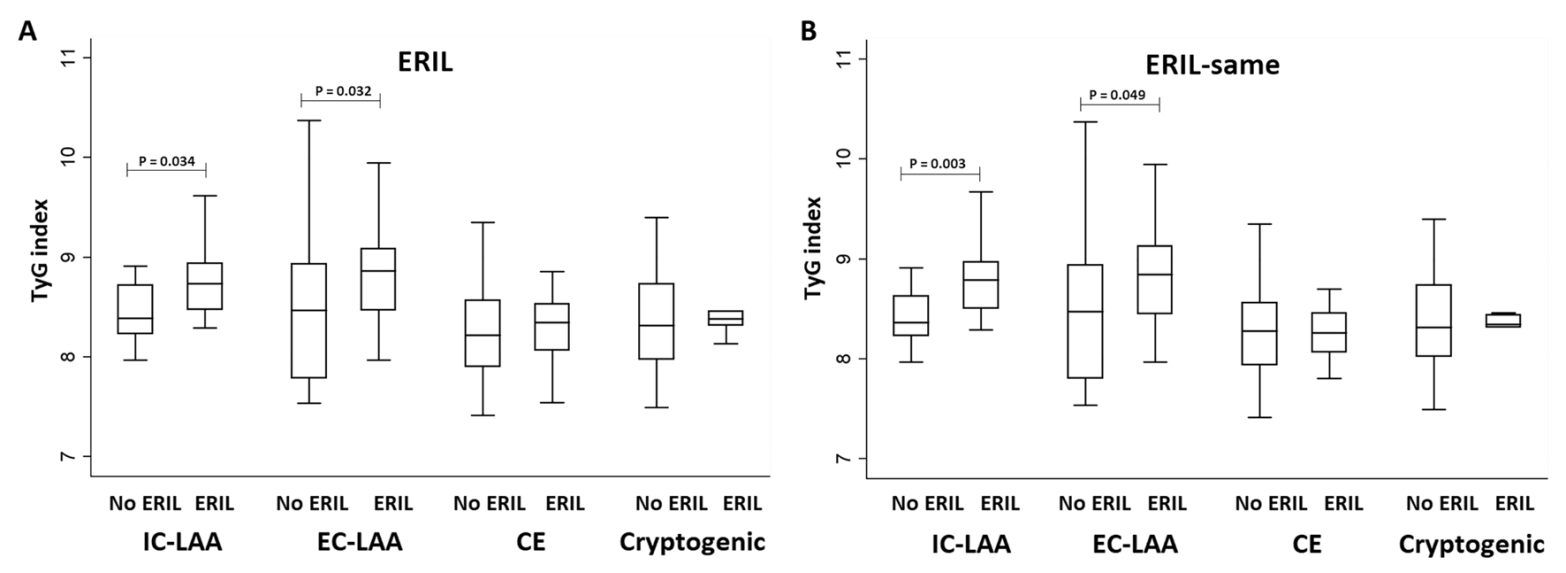
Comparisons of the TyG index values according to the presence of ERIL by stroke mechanisms. *ERIL* = early recurrent ischemic lesion, *ERIL-same* ERIL within the same vascular territory, *TyG index* triglyceride-glucose index, *IC-LAA* intracranial-large artery atherosclerosis, *EC-LAA* extracranial-large artery atherosclerosis, *CE* cardioembolism. The TyG index values show statistically significant differences in the presence or absence of ERIL only in patients in the IC-LAA (*P* = 0.034) and EC-LAA (*P* = 0.032) groups **(A)**. This difference is observed in the same pattern in ERIL-same **(B)**, but not clear in ERIL-different.

Comparing the stroke mechanisms, ERIL had a higher incidence in the IC-LAA and EC-LAA groups than in other groups, but the difference was not statistically significant (*P* = 0.115). Interestingly, however, ERIL was closely associated with different stroke mechanisms depending on the lesion location as ERIL-same was significantly detected more in the IC-LAA and EC-LAA groups (*P* = 0.001), while ERIL-different was significantly associated with the CE group (*P* = 0.016). Patients in the IC-LAA and EC-LAA groups had significantly higher TyG index (*P* < 0.001) and TG/HDL ratio (*P* < 0.001, Table 3). When we analyzed the relationship between the TyG index and ERIL based on stroke mechanism, only the patients with ERIL from the IC-LAA (*P* = 0.034) and EC-LAA (*P* = 0.032) groups showed significantly higher TyG index values than patients without ERIL (Fig. 1). In both the IC-LAA and EC-LAA groups, the association between the TyG index and ERIL was maintained when it occurred in the same vascular territory (ERIL-same) but was lost if it occurred in different vascular territory (ERIL-different).

**Table 3:** Comparison of characteristics of patient groups according to stroke mechanism.

	IC-LAA (n = 41)	EC-LAA (n = 44)	CE (n = 56)	Cryptogenic (n = 35)	*P* value
ERIL	22 (53.7)	21 (47.7)	21 (37.5)	10 (28.6)	0.115
ERIL-same	20 (48.8)	20 (45.5)	10 (17.9)	7 (20.0)	0.001
ERIL-different	2 (4.9)	1 (2.3)	11 (19.6)	3 (8.6)	0.016
Age	70 [68–76]	70 [69–73]	75 [72–82]	70 [66–75]	0.073
Sex	21 (51.2)	33 (75.0)	26 (46.4)	23 (65.7)	0.019
Visit time	1 [0–2]	0 [0–2]	0 [0–1]	0 [0–1]	0.098
Follow-up MRI time	2 [2–4]	2 [2–5]	3 [3, 4]	3 [2–5]	0.224
Hypertension	25 (61.0)	33 (75.0)	37 (66.1)	27 (77.1)	0.348
Smoking	14 (34.1)	18 (40.9)	10 (17.9)	10 (18.6)	0.077
Initial NIHSS score	5 [2–11]	5 [2–13]	6 [3–16]	3 [1–9]	0.031
White blood cell	7.56 [6.93–8.80]	8.70 [6.33–9.48]	6.78 [6.09–7.73]	7.85 [7.12–10.10]	0.136
High-sensitivity CRP	0.29 [0.07–0.50]	0.27 [0.11–0.42]	0.30 [0.12–0.53]	0.17 [0.08–0.50]	0.601
TG/HDL ratio	2.79 [2.34–3.45]	2.75 [2.09–3.73]	1.77 [1.57–1.88]	1.75 [1.23–2.32]	< 0.001
TyG index	8.58 [8.44–8.80]	8.62 [8.43–8.94]	8.28 [8.13–8.46]	8.33 [8.18–8.56]	< 0.001
Recanalization	7 (17.1)	0 (0.0)	22 (39.3)	5 (14.3)	< 0.001

*IC-LAA* intracranial large artery atherosclerosis, *EC-LAA* extracranial large artery atherosclerosis, *CE* cardioembolic, *ERIL* early recurrent ischemic lesion, *ERIL-same* ERIL within the same vascular territory, *ERIL-different* ERIL within different vascular territory, *MRI* magnetic resonance imaging, *NIHSS* National Institutes of Health Stroke Scale, *CRP* C-reactive protein, *TG* triglyceride, *HDL* high-density lipoprotein, *TyG* index = triglyceride-glucose index.

In this study, the ERIL group had a significantly worse prognosis than the no ERIL group (Table 4), experiencing more frequent END (35.1% versus 18.6%, *P* = 0.013), and had higher discharge NIHSS (2 [0–8] versus 6 [2–16], *P* < 0.001) and modified Rankin Scale (2 [1–3] versus 2 [2–5], *P* < 0.001) scores.

**Table 4:** Differences in subsequent prognosis between patients with and without early recurrent ischemic lesions.

	No ERIL (n = 102)	ERIL (n = 74)	*P* value
END, n (%)	19 (18.6%)	26 (35.1%)	0.013
Discharge NIHSS score [IQR]	2 [0–8]	6 [2–16]	< 0.001
**Discharge mRS, n (%)**	2 [1–3]	2 [2–5]	< 0.001
Good (0–2)	67 (65.7%)	39 (52.7%)	0.082
Poor (3–6)	35 (34.3%)	35 (47.3%)	
Hospitalization duration, d [IQR]	9 [7–13]	12 [8–16]	0.120

*ERIL* early recurrent ischemic lesion, *END* early neurological deterioration, *NIHSS* National Institutes of Health Stroke Scale, *mRS* modified Rankin Scale.

## Discussion

In this study, we found that the TyG index was closely associated with ERIL, especially ERIL-same, in patients with AIS. This association was pronounced in the IC-LAA and EC-LAA groups, indicating that the TyG index primarily affects atherosclerotic lesions, resulting in further recurrence of radiological lesions.

The TyG index was initially designed as a chronic IR marker^8,15^. Thus, the close association between the TyG index and ERIL may have been due to several pathological conditions occurring in the chronic IR state such as metabolic diseases, subclinical inflammation, and endothelial dysfunction^9–11,21^. In our data, the TyG index was closely related to inflammatory markers and the TG/HDL ratio, another IR marker^22^, implying that chronic IR can affect the occurrence of ERIL. Although the TG/HDL ratio was not significantly associated with ERIL, it showed a close tendency. On the other hand, the TyG index may possibly be an indicator of the compound influence of glucose and TG^20^, since hyperglycemia and hypertriglyceridemia are known to worsen the acute prognosis of ischemic stroke^18,19^. Therefore, we propose a future prospective study to analyze the relationship of ERIL incidence and the changes in the TyG index during the initial 7 days.

Of the four stroke mechanisms, ERIL had the highest incidence in the IC-LAA and EC-LAA groups, with most being classified as ERIL-same. This is thought to have been caused by the relevant atherosclerotic lesions^2,23^. In addition, the TyG index was closely related to ERIL only in the IC-LAA and EC-LAA groups. Glucose, TG, and IR are known to be closely linked to the formation of atherosclerosis and the plaque instability^10,24–26^. Indeed, both the TyG index and TG/HDL ratio were elevated in the IC-LAA and EC-LAA groups (Table 4). Therefore, it is possible that the instability of the underlying atherosclerotic plaques led to (1) artery-to-artery embolism, (2) stepwise progression from near occlusion to complete occlusion, and (3) hemodynamic failure, resulting in the occurrence of ERIL^20,27^. Consistent with the findings of previous studies, patients with stroke by CE or cryptogenic mechanism had a low frequency of ERIL^23^. Also, in these groups, the TyG index was not statistically associated with ERIL. In summary, the TyG index appeared to be involved in the recurrence of lesions caused by atherosclerosis and was irrelevant to proximal embolic sources.

The authors found interesting findings on the possible mechanism of ERIL through recanalization. Since ERIL is based on radiological recurrence independent of clinical symptoms, the breakup of an index embolus during recanalization may cause it^2^. In our data, recanalization was found most in the CE group, but not in the EC-LAA group (Table 4)^23^. Among those with observed recanalization, ERIL occurred in 6 (85.7%) patients in the IC-LAA group, 12 (54.5%) in the CE group, and 2 (40.0%) in the cryptogenic group. Of these ERILs, 83.3%, 75.0%, and 100%, respectively, were ERIL-same. Comprehensive analysis of these results for each mechanism allows for the following interpretations. In the CE group, there seems to be much fragmentation of initial embolus without actual recurrent ischemic events as in previous studies^23^. However, since ERIL-different was found in 3 (25%) patients with recanalization, it is possible that the recurrent embolisms due to the cardiac source also influenced the occurrence of ERIL. Recanalization did not occur in the EC-LAA group, suggesting that ERIL is caused only by actual recurrent ischemic events such as artery-to-artery embolism or hemodynamic failure^28^. The IC-LAA group had a non-negligible recanalization rate and resulting ERIL-same incidence. The origin of thrombi causing recanalization may be in situ thrombosis of relevant vascular lesions or embolism from proximal sites. In any case, it seems clear that IC-LAA occurs with more complex mechanisms than EC-LAA in both index lesion and ERIL^28^. TyG index values did not differ between the patients with and without recanalization. Unlike the actual ischemic recurrence occurring in atherosclerotic lesions, the TyG index did not appear to be involved in this silent recurrence.

Some caveats should be considered when interpreting our findings. First, since this was a retrospective cross-sectional study, we could only reveal the association between the TyG index and ERIL and could not prove a causal relationship. Second, selection bias during the process of selecting the study population should be considered since we excluded patients who previously used glucose- and lipid-lowering agents. Though this might lead to the said bias, we decided to exclude the complex effects of the type, dose, and timing of drug administration that could directly affect the TyG index value itself during acute periods. Third, to evaluate ERIL, we included only patients who underwent follow-up MRI within 7 days of admission. Follow-up MRI is often taken when clinical worsening occurs. In fact, in our study population, a higher frequency of 7-day clinical worsening (27.8%) was observed compared to patients with general stroke. Although this was not statistically influential, it is something to consider in interpreting our results. Finally, since our study dealt with the acute period of ischemic stroke, the effect of post-stroke hyperglycemia should also be considered.

We had demonstrated that the TyG index was associated with ERIL in patients with AIS, which was strongest when new lesions occurred in the same vascular territory as the index stroke lesion caused by atherosclerosis. Therefore, close monitoring of glucose and TG levels is required during the acute period. However, whether acute care of glucose and TG levels actually works should be determined in further prospective studies.

## Methods

### Study population

This was a retrospective cross-sectional study based on a consecutive stroke registry at a large medical center in Korea (Seoul Metropolitan Government-Seoul National University Boramae Medical Center). From this registry, we included AIS patients who underwent follow-up magnetic resonance imaging (MRI) within 7 days of admission between January 2010 and December 2016^29^. Then, the patients with the following conditions were excluded: (1) delay of > 7 days from symptom recognition to admission, (2) received diagnostic or therapeutic neuro-intervention procedures, or (3) previous use of glucose- or lipid-lowering agents before measurements^5,20^. Similar to the previous studies focusing on ERIL, we also excluded patients with the following stroke mechanisms: (1) small vessel occlusion (due to the low incidence of ERILs); (2) other determined; and (3) more than two mechanisms^23^. However, in contrast with previous studies, we included patients with cryptogenic stroke because of the recently emerging importance of cryptogenic stroke, including the embolic stroke of undetermined source. Finally, a total of 176 patients with ischemic stroke were included in the final analyses.

This study was approved by the Institutional Review Board of the Seoul Metropolitan Government-Seoul National University Boramae Medical Center (IRB number: 06-2020-0043). The requirement to obtain informed consent from the participants was waived by the Institutional Review Board because this was designed as a retrospective study using anonymous and de-identified information. All experiments were performed in accordance with the Declaration of Helsinki and relevant guidelines and regulations. All data related to this study are included in the main text and Supplemental Materials.

### Demographic, clinical and laboratory assessment

For the demographic and clinical factors, we assessed age, sex, hypertension, atrial fibrillation, current smoking, stroke mechanism, initial stroke severity, and systolic and diastolic blood pressures^21,29^. The stroke mechanism was determined according to the Trial of ORG 10172 in Acute Stroke Treatment classification^30^, and the study population was divided into four groups as follows: intracranial-large artery atherosclerosis (IC-LAA), extracranial-large artery atherosclerosis (EC-LAA), cardioembolism (CE), and cryptogenic^23^. IC-LAA was diagnosed when there was symptomatic intracranial atherosclerosis (occlusion or ≥ 50% stenosis) without evidence of EC-LAA or CE^23,31^. EC-LAA was defined as having symptomatic extracranial atherosclerosis without IC-LAA or CE^23,32^. Initial stroke severity was rated daily using the National Institutes of Health Stroke Scale (NIHSS) score from admission to discharge by trained neurologists not involved in this study.

Laboratory examinations were performed after 12 h of overnight fasting, including glucose profiles, lipid profiles, white blood cell (WBC) counts, and high-sensitivity C-reactive protein^20^. The TyG index was calculated using the formula of “the log scale of [fasting TG (mg/dL) x fasting glucose (mg/dL)/2]^15,22^. We also calculated the TG/high-density lipoprotein (HDL) cholesterol ratio as another IR marker by dividing the absolute TG levels by absolute HDL cholesterol levels^22^.

### Radiological assessment

**Figure 2:**
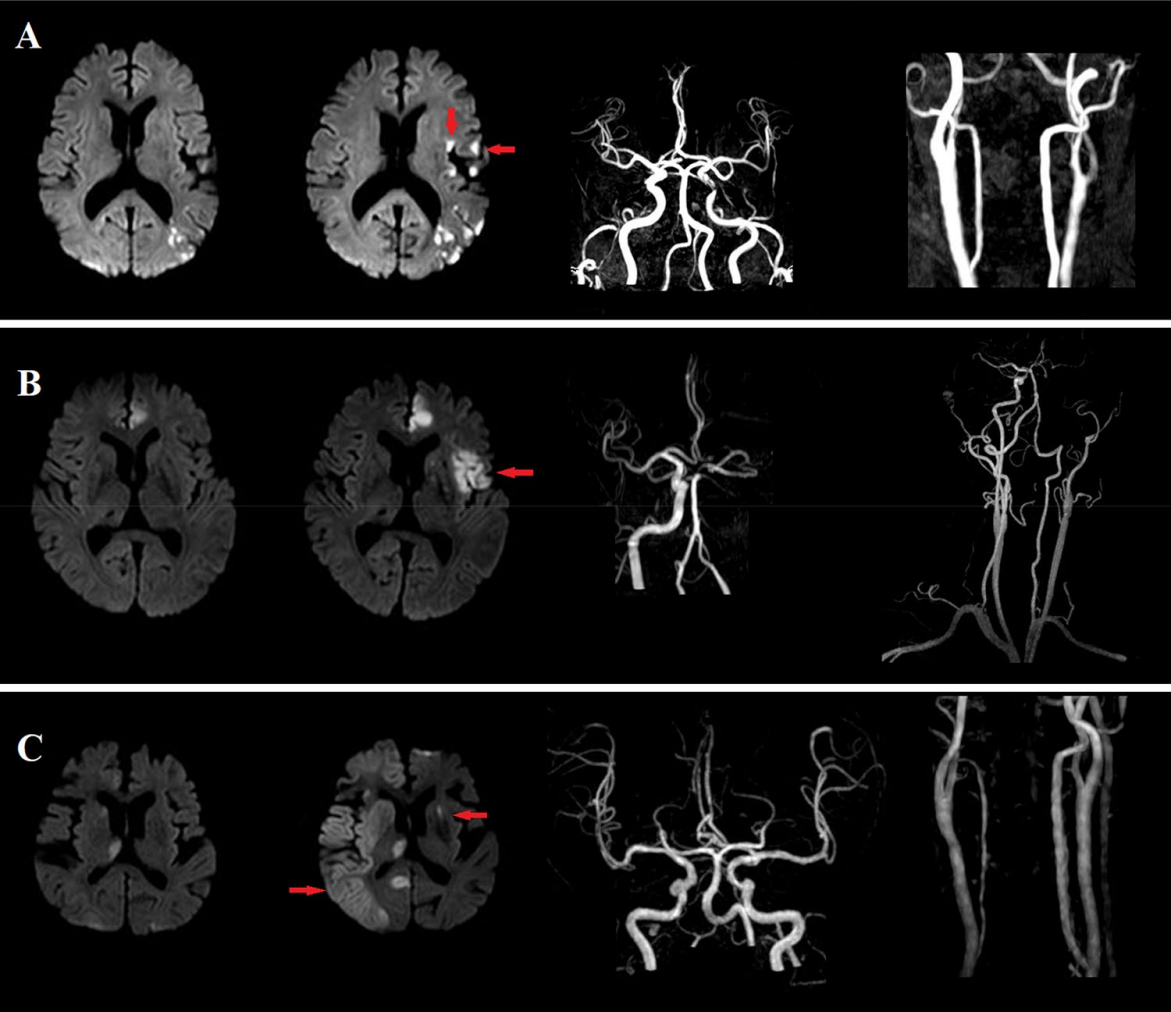
Representative cases of ERIL-same and ERIL-different. **(A)** This patient belongs to the IC-LAA group, and new lesions on follow-up MRI are occurring in the same vascular territory where the index lesion belongs: ERIL-same. **(B)** In this patient belongs to the EC-LAA group, a new lesion is detected in the left proximal ICA area, a relevant vascular territory: ERIL-same. **(C)** In this patient from the CE group, follow-up MRI lesions are detected outside the vascular territory of the index lesion: ERIL-different.

All participants underwent brain MRI and magnetic resonance angiography (MRA) within 24 h of admission using a 3.0-T MR scanner (Achieva 3.0 T; Philips, Eindhoven, the Netherlands). ERIL is a new DWI lesion occurring outside the initial symptomatic lesion area and not visible in the initial MRI^2^. The enlargement of the initial DWI lesion was not considered as ERIL^2,5^. Based on previous studies, we classified ERIL into two groups: ERIL-same and ERIL-different (Fig. 2)^23^. It was classified as ERIL-same if it occurred in vascular territories same as the index lesions, while it was classified as ERIL-different if it occurred in different vascular territories or cerebral circulation^23^. If ERIL-same and ERIL-different were detected simultaneously in a patient, this patient was classified as ERIL-different group^23^. ERIL is based on radiological findings without consideration of the accompanying clinical symptoms. Thus, some may have possibly resulted from an index cause (e.g., the breakup of index embolus)^2^. To identify the effect of these conditions, we included recanalization as a variable^23^. Recanalization was evaluated based on the initial and follow-up MRA and included both partial and complete recanalization^23^. All radiological assessments were performed by two appropriately trained neurologists (K.-W.N. and H.-M.K.), and disagreements were resolved through discussions with a third rater (Y.-S.L.).

### Statistical analysis

To evaluate the possible predictors of ERIL, univariate analyses were performed using the Student’s *t*-test or Mann–Whitney *U*-test for continuous variables and the chi-squared test or Fisher’s exact test for categorical variables. Along with the stroke mechanism, the variables with *P* < 0.10 were introduced into the multivariable logistic regression analysis. Considering the composition of the TyG index formula, both glucose and lipid profiles were not introduced simultaneously with the TyG index into the multivariable analysis^15^. As a sensitivity analysis, we conducted an additional multivariable analysis using the TG/HDL ratio as another marker of IR^22^.

In previous studies, ERIL had different incidences depending on the stroke mechanism^23^. As such, to confirm these findings, the characteristics of the patient group, including the frequency of ERIL and the level of TyG index, were compared according to stroke mechanism using the chi-squared test and the Kruskal–Wallis test. Furthermore, we compared the TyG index values between patients with and without ERIL in the four groups to analyze its effect on the occurrence of ERIL by stroke mechanism. All statistical analyses were performed using SPSS version 23.0 (IBM Corp., Armonk, NY, USA). In this study, all variables with *P* < 0.05 were considered statistically significant.

## Supplementary Information

Supplementary Information.
