# Supplementary material for: High triglyceride-glucose index is associated with early recurrent ischemic lesion in acute ischemic stroke
Source: Sci Rep. 2021 Jul 28;11:15335. doi: 10.1038/s41598-021-94631-5 (PMC8319389; doi:10.1038/s41598-021-94631-5)
Supplement: Supplementary file 1 — Supplementary Information. [file 41598_2021_94631_MOESM1_ESM.pdf]

Supplemental information

Supplementary Table 1. Baseline characteristics of the study population (n = 176)

**Demographic & clinical factors**	
Age, y [SD]	71 ± 13
Sex, male, n (%)	103 (58.5)
Visit time, d [SD]	1 ± 1
Follow-up MRI time, d [SD]	3 ± 2
Hypertension, n (%)	122 (69.3)
Atrial fibrillation, n (%)	53 (30.1)
Smoking, n (%)	52 (29.5)
Stroke mechanisms, n (%)	
Intracranial-LAA	41 (23.3)
Extracranial-LAA	44 (25.0)
Cardioembolic	56 (31.8)
Cryptogenic	35 (19.9)
Initial NIHSS score [SD]	7 ± 7
Systolic BP, mmHg [SD]	155 ± 31
Diastolic BP mm Hg [SD]	85 ± 18
**Laboratory factors**	
HbA1c, % [SD]	6.2 ± 1.3
Fasting glucose, mg/dL [SD]	112 ± 41
Total cholesterol, mg/dL [SD]	183 ± 43
LDL cholesterol, mg/dL [SD]	109 ± 38
HDL cholesterol, mg/dL [SD]	43 ± 11
Triglyceride, mg/dL [SD]	100 ± 54
White blood cell, x 10^3/uL [SD]	8.27 ± 3.08
High-sensitivity CRP, mg/dL [SD]	1.27 ± 3.60
TG/HDL ratio [SD]	2.60 ± 1.73
TyG index [SD]	8.48 ± 0.55
**Radiological factors**	
ERIL, n (%)	74 (42.0)
ERIL-same, n (%)	57 (32.4)
ERIL-different, n (%)	17 (9.7)
Recanalization, n (%)	34 (19.3)

MRI = magnetic resonance imaging, LAA = large artery atherosclerosis, NIHSS = National Institutes of Health Stroke Scale, BP = blood pressure, LDL = low-density lipoprotein, HDL = high-density lipoprotein, CRP = C-reactive protein, TG = triglyceride, TyG index = triglyceride-glucose index, ERIL = early recurrent ischemic lesion, ERIL-same = ERIL within the same vascular territory, ERIL-different = ERIL within different vascular territory

Supplementary Table 2. Univariate linear regression analysis between the TyG index and clinical, laboratory, and radiological parameters

	β (95% CI)	*P* value
Age	-0.006 (-0.012 to 0.000)	0.054
Sex	0.108 (-0.058 to 0.275)	0.201
Hypertension	0.150 (-0.027 to 0.327)	0.096
Atrial fibrillation	-0.333 (-0.505 to -0.160)	< 0.001
Smoking	0.074 (-0.106 to 0.254)	0.420
Stroke mechanisms		
IC-LAA	0.260 (0.069 to 0.451)	0.008
EC-LAA	0.242 (0.055 to 0.429)	0.011
Cardioembolic	-0.328 (-0.498 to -0.158)	< 0.001
Cryptogenic	-0.130 (-0.336 to 0.075)	0.212
Initial NIHSS score	-0.003 (-0.015 to 0.009)	0.650
Systolic BP, mmHg	0.002 (-0.001 to 0.005)	0.168
Diastolic BP, mmHg	0.002 (-0.003 to 0.007)	0.418
White blood cell	0.050 (0.025 to 0.076)	< 0.001
High-sensitivity CRP	0.022 (-0.001 to 0.044)	0.059
TG/HDL ratio	0.241 (0.209 to 0.272)	< 0.001
Recanalization	-0.186 (-0.393 to 0.021)	0.078
ERIL	0.245 (0.082 to 0.408)	0.003
ERIL-same	0.330 (0.161 to 0.499)	< 0.001
ERIL-different	-0.146 (-0.424 to 0.132)	0.302

TyG index = triglyceride-glucose index, IC-LAA = intracranial large artery atherosclerosis, EC-LAA = extracranial large artery atherosclerosis, NIHSS = National Institutes of Health Stroke Scale, BP = blood pressure, CRP = C-reactive protein, ERIL = early recurrent ischemic lesion, ERIL-same = ERIL within the same vascular territory, ERIL-different = ERIL within different vascular territory

Supplementary Table 3. Univariate and multivariable analyses of possible predictors for ERIL within the same vascular territory (ERIL-same)

	Crude OR (95% CI)	*P*-value	Adjusted OR (95% CI)	*P*-value
Age, y	1.01 [0.99-1.04]	0.387	1.03 [1.00-1.06]	0.044
Sex, male	1.33 [0.70-2.55]	0.388	…	…
Visit time, d	1.11 [0.89-1.37]	0.358	…	…
Follow-up MRI time, d	1.01 [0.88-1.16]	0.872	…	…
Hypertension	0.83 [0.42-1.64]	0.598	…	…
Atrial fibrillation	0.38 [0.17-0.82]	0.014	…	…
Smoking	1.02 [0.51-2.04]	0.955	…	…
Stroke mechanisms		0.001		0.001
IC-LAA	2.52 [1.23-5.18]	0.012	3.17 [1.02-9.89]	0.047
EC-LAA	2.14 [1.06-4.33]	0.034	4.05 [1.30-12.56]	0.016
Cardioembolic	0.34 [0.16-0.73]	0.006	0.39 [0.11-1.43]	0.156
Cryptogenic	0.46 [0.19-1.12]	0.085	Ref	Ref
Initial NIHSS score	1.02 [0.98-1.07]	0.368	…	…
Systolic BP, mmHg	1.00 [0.99-1.01]	0.629	…	…
Diastolic BP mm Hg	1.00 [0.99-1.02]	0.738	…	…
HbA1c, %	1.03 [0.80-1.32]	0.841	…	…
Fasting glucose, mg/dL	1.01 [1.00-1.01]	0.081	…	…
Total cholesterol, mg/dL	1.01 [1.00-1.02]	0.005	…	…
LDL cholesterol, mg/dL	1.01 [1.00-1.02]	0.006	…	…
HDL cholesterol, mg/dL	1.00 [0.97-1.03]	0.847	…	…
Triglyceride, mg/dL	1.01 [1.00-1.02]	0.005	…	…
White blood cells, x 10^3/uL	1.01 [0.91-1.12]	0.811	…	…
High-sensitivity CRP, mg/dL	0.95 [0.85-1.07]	0.413	…	…
TG/HDL ratio	1.27 [1.04-1.54]	0.019	…	…
TyG index	3.10 [1.65-5.83]	< 0.001	2.84 [1.40-5.78]	0.004
Recanalization	2.19 [1.02-4.71]	0.045	8.53 [2.79-26.07]	< 0.001

ERIL = early recurrent ischemic lesion, MRI = magnetic resonance imaging, IC-LAA = intracranial large artery atherosclerosis, EC-LAA = extracranial large artery atherosclerosis, NIHSS = National Institutes of Health Stroke Scale, BP = blood pressure, LDL = low-density lipoprotein, HDL = high-density lipoprotein, CRP = C-reactive protein, TG = triglyceride, TyG index = triglyceride-glucose index

Supplementary Table 4. Univariate and multivariable analyses of possible predictors for ERIL within different vascular territory (ERIL-different)

	Crude OR (95% CI)	*P*-value	Adjusted OR (95% CI)	*P*-value
Age, y	1.06 [1.01-1.12]	0.011	1.02 [0.96-1.08]	0.508
Sex, male	0.26 [0.09-0.77]	0.015	0.63 [0.17-2.32]	0.489
Visit time, d	0.67 [0.38-1.17]	0.159	…	…
Follow-up MRI time, d	1.12 [0.91-1.36]	0.283	…	…
Hypertension	1.07 [0.36-3.20]	0.905	…	…
Atrial fibrillation	5.11 [1.78-14.67]	0.002	…	…
Smoking	0.13 [0.02-1.03]	0.053	0.28 [0.03-2.69]	0.269
Stroke mechanisms		0.041		0.213
IC-LAA	0.41 [0.09-1.87]	0.250	0.42 [0.06-2.89]	0.377
EC-LAA	0.17 [0.02-1.31]	0.089	0.24 [0.02-2.52]	0.233
Cardioembolic	4.64 [1.62-13.31]	0.004	1.48 [0.35-6.39]	0.596
Cryptogenic	0.85 [0.23-3.14]	0.808	Ref	Ref
Initial NIHSS score	1.10 [1.02-1.17]	0.008	1.07 [0.99-1.15]	0.095
Systolic BP, mmHg	1.01 [0.99-1.02]	0.299	…	…
Diastolic BP mm Hg	1.01 [0.98-1.04]	0.471	…	…
HbA1c, %	0.60 [0.27-1.32]	0.204	…	…
Fasting glucose, mg/dL	1.00 [0.98-1.01]	0.482	…	…
Total cholesterol, mg/dL	0.99 [0.98-1.00]	0.120	…	…
LDL cholesterol, mg/dL	0.99 [0.98-1.01]	0.263	…	…
HDL cholesterol, mg/dL	0.98 [0.94-1.03]	0.518	…	…
Triglyceride, mg/dL	0.99 [0.98-1.01]	0.326	…	…
White blood cell, x 10^3/uL	0.98 [0.83-1.16]	0.778	…	…
hs-CRP, mg/dL	1.08 [0.99-1.19]	0.100	…	…
TG/HDL ratio	0.94 [0.68-1.29]	0.691	…	…
TyG index	0.59 [0.22-1.60]	0.301	…	…
Recanalization	1.32 [0.40-4.35]	0.644	…	…

ERIL = early recurrent ischemic lesion, MRI = magnetic resonance imaging, IC-LAA = intracranial large artery atherosclerosis, EC-LAA = extracranial large artery atherosclerosis, NIHSS = National Institutes of Health Stroke Scale, BP = blood pressure, LDL = low-density lipoprotein, HDL = high-density lipoprotein, CRP = C-reactive protein, TG = triglyceride, TyG index = triglyceride-glucose index

Supplementary Table 5. Sensitivity multivariable analysis of possible predictors for ERIL using the TG/HDL ratio

	Crude OR (95% CI)	*P*-value	Adjusted OR (95% CI)	*P*-value
Age	1.03 [1.01-1.05]	0.020	1.03 [1.01-1.06]	0.015
Stroke mechanism		0.121		0.047
IC-LAA	2.90 [1.11-7.53]	0.029	2.25 [0.79-6.42]	0.129
EC-LAA	2.28 [0.89-5.86]	0.086	2.37 [0.85-6.63]	0.101
Cardoembolic	1.50 [0.60-3.73]	0.383	0.71 [0.25-2.02]	0.521
Cryptogenic	Ref	Ref	Ref	Ref
Initial NIHSS score	1.06 [1.01-1.11]	0.013	1.06 [1.01-1.11]	0.032
Recanalization	2.33 [1.09-4.99]	0.030	4.06 [1.62-10.16]	0.003
TG/HDL ratio	1.22 [1.01-1.48]	0.041	1.23 [0.99-1.53]	0.061

ERIL = early recurrent ischemic lesion, TG = triglyceride, HDL = high-density lipoprotein, IC-LAA = intracranial large artery atherosclerosis, EC-LAA = extracranial large artery atherosclerosis, NIHSS = National Institutes of Health Stroke Scale
